# The Potential of Exosomes in Allergy Immunotherapy

**DOI:** 10.3390/vaccines10010133

**Published:** 2022-01-17

**Authors:** Paul Engeroff, Monique Vogel

**Affiliations:** 1 Sorbonne Université, INSERM, Immunology-Immunopathology-Immunotherapy (i3), F-75005 Paris, France; paul.engeroff@sorbonne-universite.fr; 2 Department of Immunology, University Hospital for Rheumatology, Immunology, and Allergology, 3010 Bern, Switzerland; 3 Department of BioMedical Research, University of Bern, 3008 Bern, Switzerland

**Keywords:** Type I hypersensitivity, IgE, AIT, SIT, extracellular vesicles, vaccine, mast cells, mesenchymal stem cells

## Abstract

Allergic diseases represent a global health and economic burden of increasing significance. The lack of disease-modifying therapies besides specific allergen immunotherapy (AIT) which is not available for all types of allergies, necessitates the study of novel therapeutic approaches. Exosomes are small endosome-derived vesicles delivering cargo between cells and thus allowing inter-cellular communication. Since immune cells make use of exosomes to boost, deviate, or suppress immune responses, exosomes are intriguing candidates for immunotherapy. Here, we review the role of exosomes in allergic sensitization and inflammation, and we discuss the mechanisms by which exosomes could potentially be used in immunotherapeutic approaches for the treatment of allergic diseases. We propose the following approaches: (a) Mast cell-derived exosomes expressing IgE receptor FcεRI could absorb IgE and down-regulate systemic IgE levels. (b) Tolerogenic exosomes could suppress allergic immune responses via induction of regulatory T cells. (c) Exosomes could promote TH1-like responses towards an allergen. (d) Exosomes could modulate IgE-facilitated antigen presentation.

## 1. Allergy and Allergy Immunotherapy

Allergic diseases are a global issue as more and more people are affected by allergies [1]. Type I hypersensitivity is characterized by abnormal IgE-mediated inflammation in response to harmless antigens called allergens caused by a lack of immune tolerance coupled with the expansion of TH2 cells that drive IgE responses from B cells [2,3]. Allergen-specific IgE sensitizes mast cells and basophils by binding to the high-affinity IgE receptor FcεRI [4]. Upon secondary contact with the allergen, those cells degranulate and release inflammatory mediators [5]. Symptomatic treatment options for allergies involve down-regulation of the mediators released by mast cells or basophils (e.g., anti-histamine) or aim to down-regulate IgE levels, such as the monoclonal anti-IgE antibody Omalizumab [6]. The only disease modifying treatment available for some but not all allergies is allergen-specific immunotherapy (short AIT or SIT). AIT is a repeated immunization approach that aims to re-educate the immune system and generate tolerance towards the allergen [7,8]. Mechanistically, AIT induces regulatory T cells and B cells that are able to produce anti-inflammatory cytokines, such as IL-10 and TGF-β. This leads to suppression in TH2 responses but increased IgG4 production [9,10,11]. A novel approach in allergy immunotherapy is to boost immune responses by eliciting a non-allergic, but rather anti-viral/bacterial TH1-like response in an attempt to shift the immune response [12]. In general, it is accepted that IgG antibodies can suppress IgE-mediated effector functions by competing for the allergen epitope thus neutralizing IgE, or by ligation of inhibitory FcγRIIb receptors on mast cells/basophils [13,14,15]. FcγRIIb receptors contain immunoreceptor tyrosine-based inhibitory motif (ITIM) signaling domains that shut down FcεRI-dependent effector cell activation [16,17,18]. Additionally, FcγRs and IgG can promote the internalization of IgE thus preventing IgE-dependent activation of mast cells and basophils [19]. However, further research in this area is still required as IgG can also contribute to inflammation, depending on the IgG subclass and type of FcγR receptor involved [20]. In summary, AIT is a viable therapy that still requires improvement as it is not available for all allergens and often bears the risk of side effects caused by allergen application, which can degranulate allergic effector cells via FcεRI-displayed IgE. Therefore, there is a need for novel treatment strategies that reduce side effects while maintaining efficacy.

## 2. The Biology of Exosomes

The biology of exosomes was reviewed by Kalluri & LeBleu [21]. In brief, Extracellular vesicles (EVs) are small membrane blebs with a diameter of approximately 40 nm–1 µm released from all cell types. They are found in different fluids, such as plasma, urine, semen, bronchial fluid, and synovial fluid. Exosomes (40–160 nm) are of endosomal origin, which distinguishes them from ectosomes that bud from the surface of plasma membranes. Exosomes are surrounded by a bilayer lipid membrane composed of cholesterol, phosphatidylserines, and sphingolipids, which confer protection against proteases and RNases [22]. Exosome surface proteins include the classical exosome markers, the Tetraspanin proteins CD9, CD63, CD81, CD82, as well as adhesion molecules and the immune regulator molecules major histocompatibility complex (MHC) Class I and II [23]. Tetraspanins are involved in cell penetration, invasion, and fusion events allowing exosomes to provide their targets with molecules by transferring membrane material by fusing to target cells without the need of direct cell-cell contact. Besides their surface proteins, exosomes can carry a variety of cargo proteins. Exosomes are enriched with endosomal proteins from parent cells implicated in exosome biogenesis including ESCRT-related proteins (Alix and TSG101) and cytoplasmic proteins, such as Annexins and Rab GTPases, responsible for membrane transport and fusion of exosomes to the cell membrane. Additionally, exosomes contain heat shock proteins (e.g., HSP90, HSP70) which help peptide loading on MHC, and metabolic enzymes, such as ATPase or Glyceraldehyde-3-phosphate dehydrogenase (GAPDH) [24]. Besides their protein cargo, exosomes are loaded with cargo RNA. Different types of RNAs are enriched in exosomes including coding messenger RNA and non-coding RNAs, such as ribosomal RNA (rRNA) and miRNA. Those miRNAs can act as regulator molecules in recipient cells to alter gene expression at the post-transcriptional level by targeting mRNA transcripts [25,26]. In summary, exosomes contain a variety of molecules that are involved in key cellular functions that play a role in physiological and pathological processes [27].

## 3. The Biomedical Application of Exosomes

EVs, and specifically exosomes have been a fast-rising topic in the development of novel biomedical therapeutics due to their relevance as cellular communicators. The recent advances in engineering and application of exosomes were reviewed in depth by Perocheau et al. [28]. Of specific interest to the field of immunology is the fact that immune responses are regulated by exosomes as antigens, MHC complexes, and co-stimulatory molecules are transferred between immune cells. Furthermore, the physiological nature of exosomes as delivery agents has been viewed as a potential advantage compared to other therapeutics that can elicit anti-drug responses [29]. Other advantages include their non-toxicity, their wide distribution in biological fluids, the ability to induce functional responses in specific target cells, their variability in cargo as well as their ability to easily cross biological barriers. The fact that exosome function is dysregulated in a variety of diseases makes their direct therapeutic potential even more intriguing [30]. It is established that exosomes play a major role during various steps of cancer growth and metastasis [31]. Another example are viruses, such as Human T-cell Lymphotropic Virus Type-1 (HTLV-1) in which viral proteins packaged into EVs promote the development of inflammation and enhance viral spread in organs and peripheral blood [32]. Of interest for this review is the fact that exosomes also seem to play a role in the dysregulated communication in allergy and allergic asthma [33]. Recently, several exosome-based immunotherapeutics have entered clinical trials, demonstrating the promise of this technology. The various candidates are produced in mammalian cell culture or cow’s milk. Whereas some candidates are used unmodified others have been specifically engineered, for example by expressing the pro-inflammatory cytokine IL-12 or Stimulator of Interferon Genes (STING) which aim to promote the identification and killing of cancer cells by the immune system [34,35]. Furthermore, exosomes can be used to cross the blood-brain barrier in order to deliver siRNA for reducing the expression of genes, such as BACE which is a potential gene involved in Alzheimer’s disease [36]. Exosomes are also used as a delivery system shielding other established therapeutics from anti-drug responses by the immune system [37]. Even though exosome technology has progressed quickly in recent years, the main hurdle left is the large-scale production and purification of exosomes. Current exosome purification methods include density gradient and ultracentrifugation, chromatography or precipitation using chemicals polymers or capture by antibodies which have been compared by Konoshenko et al. [38]. In conclusion, while this new technology is still evolving, several exosome-based therapeutics are moving into clinics thus highlighting their potential as a new generation of therapeutics.

## 4. Exosomes in the Regulation of Immune Responses

The transfer of exosomes between immune cells can have a strong functional effect on immune responses and either promote, deviate or suppress immune responses [39,40,41]. Many studies have shown that dendritic cells modulate CD4+, as well as CD8+ immune responses via exosomes that carry MHCI or MHCII molecules as well as co-stimulatory molecules CD8/CD86 [42,43,44]. Interestingly, extracellular vesicles containing intact p-MHC complexes pre-loaded with antigen-derived peptide can also lead to direct antigen presentation to T cells without the need for antigen processing, this mechanism of antigen presentation is referred to as “cross-dressing” [45,46,47]. In cross-dressing, DC-derived exosomes can be recaptured by DC and can remain on the cell surface to be directly presented by DC or internalized to be reloaded on endogenous MHC-class I molecules. Besides the exosomal transfer of p-MHCII and native antigens, RNA cargo provides a fundamental mechanism for intercellular communication [25]. Donor cells package mRNA or small non-coding micro RNAs (miRNAs) into exosomes. In the exosome-receiving cell, mRNAs can be translated into proteins and miRNAs can post-transcriptionally regulate target mRNAs. The transfer of miRNA exists in immune cells as means for antigen presenting cells (APCs) communication and activation [48,49,50]. Interestingly, viruses can highjack this system as EBV-infected B cells transfer viral miRNAs to DCs that silence immune-stimulatory molecules [51]. Regulatory T cells can suppress CD4+ T cell proliferation and cytokine production by transferring miRNA via exosomes which block gene expression [52,53]. Mesenchymal stem cells (MSC) are of high interest in the treatment of inflammatory diseases as they produce anti-inflammatory exosomes that can suppress DC maturation, T cell activation and promote regulatory T cells and B cells [54,55,56,57]. Cancer cells use exosomes carrying tumor antigens or inhibitory molecules that can suppress the activation of DCs, T cells and NK cells in the tumor microenvironment [58,59,60]. Due to the high variety of immunomodulatory properties, exosomes have the potential to specifically address different types of diseases depending on immunopathology [61,62,63].

## 5. Exosomes in Allergic Sensitization and Inflammation

**Figure 1:**
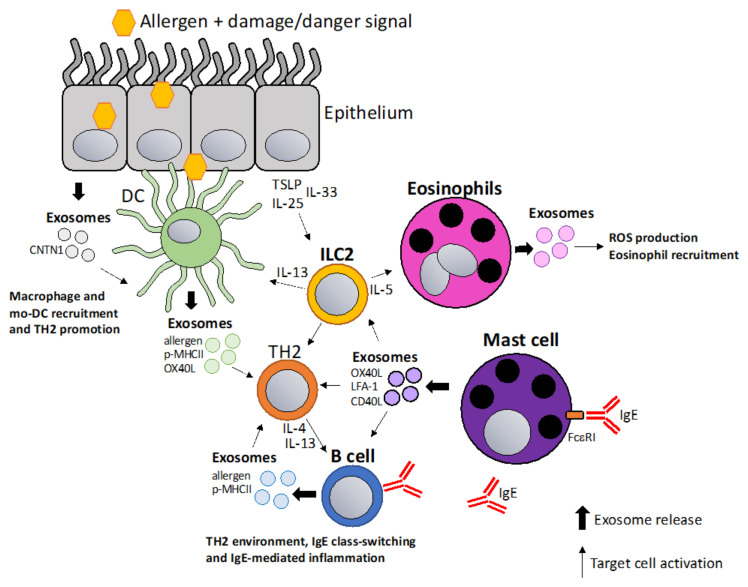
Exosomes in allergic sensitization and inflammation. Allergic sensitization is thought to be driven by allergen entry through epithelium in a damaged or inflamed environment. Epithelial cells release TSLP/IL-33/IL-25 activating DCs and ILC2 cells which initiate type 2 immunity (TH2). Additionally, epithelial cells release exosomes containing contactin-1 (CNTN1) that play a role in recruiting macrophages and mo-DCs [75]. DC-derived and B cell-derived exosomes containing allergen, p-MHCII complexes and co-stimulatory molecules can amplify the TH2 milieu [66,81]. ILC2 cells activate and recruit eosinophils via IL-5 resulting in exosome release that promote ROS production in eosinophils and further eosinophil recruitment [76]. Mast cell-derived exosomes can amplify TH2 responses by stimulating lymphocytes via co-stimulatory molecules, such as OX40L, LFA-1 and CD40L [85,86].

Exosomes play a major role in allergy including sensitization, allergen presentation and TH2 polarization, and the recruitment and activation of macrophages and eosinophils. Allergic sensitization is driven by barrier disruption of skin or lung where inflammatory signals from epithelial cells, including thymic stromal lymphopoietin (TSLP)/IL-25/IL-33, are thought to activate type 2 innate lymphoid cells (ILC2) and thus drive type 2 immunity [64,65]. Interestingly, it was shown that TSLP-activated DCs release OX40L expressing exosomes that drive CD4+ TH2 proliferation and differentiation [66]. Exosomes are involved in asthmatic inflammation which has been reviewed in depth by Cañas et al. [67]. MicroRNAs seem to play a significant role in the asthmatic process, which was supported by findings that miRNA is differentially expressed in the sputum of asthma patients [68,69]. Another interesting study showed differences in 24 exosomal miRNAs in bronchoalveolar fluids (BAL) of allergic versus asthmatic patients [70]. The miRNA-17-92 cluster (miRNA-17-5p, miRNA-17-3p, miRNA-18a, miRNA-19a, miRNA-19b, miRNA-20a, and miRNA-92-1) was shown to be an important general regulator of T cell biology [71] and among the different miRNAs in the cluster, miR-19 is specifically upregulated in CD4+ T cells from asthmatic patients compared to healthy individuals [72]. Like the miR-17-92, the miR-23 cluster plays a role in T cell function and in particular in controlling TH2 differentiation by targeting IL-4 and GATA3 [73]. Upon allergen exposure, exosomes released from epithelial cells induce the proliferation and the chemotaxis of macrophages during asthmatic inflammation [74]. Recently, a study showed that in epithelial exosomes, contactin-1 (CNTN1) is involved in the activation and recruitment of monocyte-derived dendritic cells and T-cell responses in allergic asthma [75]. Likewise, eosinophil-derived exosomes promote eosinophil migration, augment adhesion by a specific increase of adhesion molecules, such as ICAM-1 and induce reactive oxygen species (ROS) and nitric oxide (NO) production in an autocrine fashion [76]. Additionally, this leads to alveolar epithelial cell (AEC) death, delay wound repair and increase airway smooth muscle cell proliferation which causes airway obstruction and tissue remodeling [77]. Exosome production is also increased by airway allergen exposure as it was shown that PBMCs from house dust mites (HDM) allergic patients produce higher numbers of exosomes in response to HDM re-stimulation and HDM-induced exosomes were also shown to contain altered cargo/properties than exosomes produced in unstimulated PBMCs [78,79]. An interesting report showed that DCs are able to package native cat allergen Fel d 1 into exosomes [80]. B cell-derived exosomes were reported to carry processed birch allergen Bet V 5 peptide/MHCII complexes that can stimulate proliferation, IL-5, and IL-13 production from BET v 1 specific T cells lines [81]. Hence, even though detailed mechanistic studies are still required to better understand the exact role of exosomes in allergy, they have been shown to be involved in a number of key allergic processes due to their immunomodulatory function and are thus attractive candidates for the development of novel therapeutics in allergic diseases (Figure 1).

## 6. Potential Exosome-Based Therapeutic Approaches in Allergy Immunotherapy

### 6.1. The Therapeutic Potential of Mast Cell-Derived Exosomes

**Figure 2:**
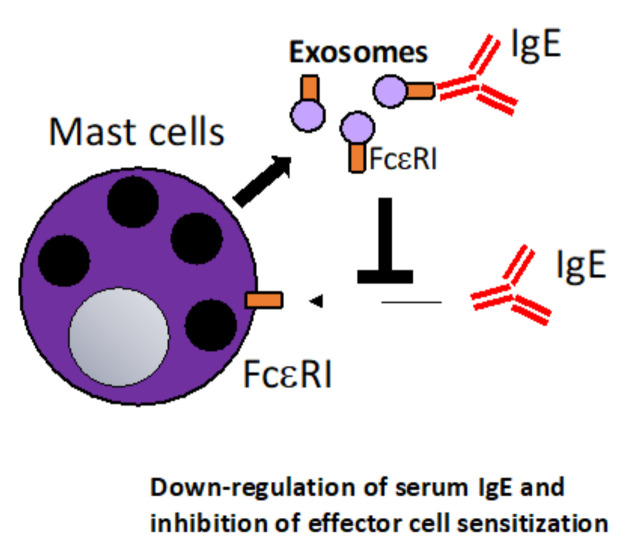
Mast cell (MC)-derived exosomes that neutralize serum IgE. MC release exosomes that express the high-affinity IgE receptor FcεRI on the surface. The exosomes can thus act as a “decoy receptor”, negatively regulate binding of IgE to MC surface FcεRI. This mechanism could be harnessed in that MC-derived exosomes or exosomes engineered to express FcεRI could be used to systemically down-regulate serum IgE levels in allergic patients [89].

Mast cells (MC) are in many ways the quintessential cell in allergy and thus also of high interest in allergy immunotherapy. MCs are located at barrier sites in the skin, lung and gut and are involved in allergic sensitization, inflammation but also resolution and tolerance. MCs express the high affinity receptor for IgE, FcεRI which upon cross-linking of the FcεRI-bound IgE by allergen induce a signal pathway leading to the release of preformed granules [82]. Likewise, MCs are a source of exosomes from the endosomal compartment which are released by exocytosis of the cells and like other immune cells, MCs can shuttle MHC molecules, RNA and chaperones that are able to modulate immune responses [83]. It was reported that mast cell-derived exosomes containing MiR103a-3p enhance IL-5 production from ILC2s [84]. Furthermore, MCs can promote TH2 immune responses by OX40L expressing exosomes that interact with OX40 on T cells and can regulate lymphocyte activation via molecules, such as CD86, LFA-1 and CD40L [85,86]. An interesting study suggested that MCs regulate their own differentiation via exosomes by communicating with blood CD34+ progenitor cells [87]. Furthermore, it was reported that mast cell-derived exosomes can induce functional maturation of DCs for efficient antigen cross-presentation to T cells [88]. A specific interest for allergy immunotherapy is the fact that mast cell-derived exosomes express FcεRI [83]. It was shown in an elegant study that mast cell-derived exosomes can neutralize IgE via surface displayed FcεRI [89]. As mentioned above, anti-IgE therapy is an established approach in the treatment of allergy [90]. Using IgE-neutralizing mast cell-derived exosomes that reduce systemic IgE levels and thus down-regulate systemic allergic sensitization could be a novel approach (Figure 2). Alternatively, FcεRI could also be engineered to be expressed on exosomes from other cell types than mast cells. FcεRI expressing exosomes could also be combined with allergen-specific approaches to gain additive effects of allergen-specific immune stimulation and simultaneous down-regulation of IgE levels. Thus, mast cell-derived exosomes specifically or exosomes engineered to express FcεRI are very attractive candidates for the engineering of novel therapeutic strategies in allergy due to their effect as an anti-IgE agent.

### 6.2. “Tolerosomes” for the Suppression of Allergic TH2 Responses

**Figure 3:**
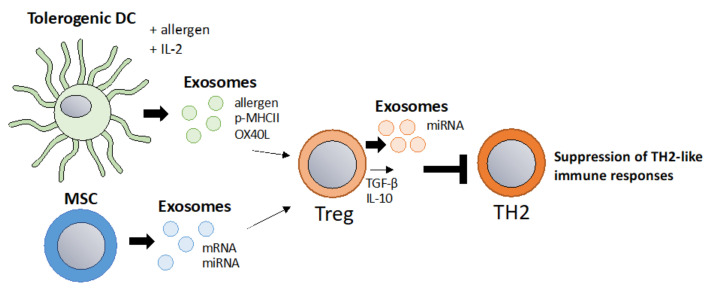
Tolerosomes derived from DCs, IEC or MSC to induce allergen tolerance via Tregs. A main goal in allergy immunotherapy is the induction of regulatory T cells. Exosomes derived from IL-2 primed tolerogenic DCs were shown to promote regulatory T cells [98]. Additionally, MSC derived exosomes promote regulatory T cells [96]. As Tregs suppress TH2 responses via IL-10, TGF-b and via exosomes, Treg-derived exosomes themselves could be harnessed to suppress TH2 responses [53].

A key concept of allergy immunotherapy is the generation of tolerance towards the allergen [91]. Tolerance-inducing exosomes also referred to as “tolerosomes” can be produced by gut epithelial cells and promote regulatory T cells and can suppress immune responses in an MHC dependent fashion [92,93]. It was demonstrated that OVA-fed mice display elevated levels of serum tolerosomes that suppress allergic sensitization and protect from allergic asthma in a murine model [94]. Another interesting study showed that exosomes isolated from BAL fluid of tolerized donor mice suppress allergic inflammation in recipient mice by promoting allergen-specific regulatory T cells that can suppress immune responses altogether thus limiting the production of IgE and airway inflammation [95]. MSC-derived exosomes have been shown to promote tolerogenic DCs by reducing maturation and production of inflammatory cytokines [57]. This concept was also confirmed in allergy as MSC-derived exosomes suppress allergic rhinitis in a murine model and human MSC-derived exosomes suppress Th2 differentiation via the miR-146a-5p/SERPINB2 axis [56,96]. In a different study, it was shown that MSC-derived exosomes alleviate atopic dermatitis [97]. Interestingly, regulatory DCs stimulated with IL-2 and antigen produce exosomes carrying IL-2 and p-MHCII complexes which promote Tregs and suppress allergic inflammation in a murine model of food allergy [98]. This concept fits into the strategy of expanding T regs via IL-2, which has been a promising and fast-evolving field, including in allergy immunotherapy [99,100]. It is generally accepted that T reg released IL-10 and TGF−β are important for the establishment of allergen tolerance [101]. Additionally, Tregs themselves have been reported to suppress immune responses via exosomes which could be applied in a similar fashion [52,53]. Hence, exosomes from tolerogenic DCs, MSCs, or T regs could be used for the inductions of T regs and tolerance in allergy immunotherapy (Figure 3).

### 6.3. Exosomes as a Vaccination Approach to Boost Th1 Responses towards Allergens

**Figure 4:**
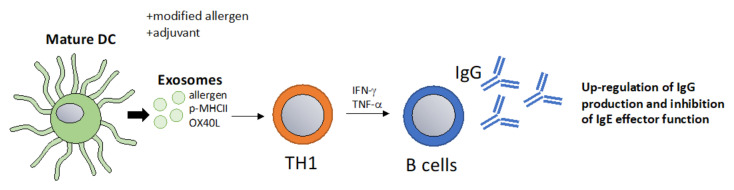
DC-derived exosomes as a vaccination approach that boost protective IgG responses. Exosomes derived from mature DCs pulsed with allergens promote T cell and B cell activation resulting in a classical TH1 type immune response with increased IgG titers [107]. High IgG titers inhibit IgE-mediated effector functions and thus allergic inflammation. This method could be optimized in multiple ways for example by the addition of specific adjuvants or by packaging allergens that are modified to be more immunogenic.

Recent approaches to improve AIT have focused on optimizing high allergen-specific IgG titers without activating sensitized allergic effector cells [102]. The properties of allergens can be changed in a way that they become more immunogenic for example by using adjuvants, such as aluminium hydroxide (Al(OH)3), microcrystalline tyrosine (MCT), monophosphoryl lipid A (MPLA) and calcium phosphate (CaP) [102]. At the same time, immunization approaches aim to deliver allergens in a way that FcεRI cannot be cross-linked on allergic effector cells, causing allergic reactions or anaphylaxis. Examples of strategies to reduce allergen reactogenicity while maintaining immunogenicity include peptide immunotherapy or intra-lymphatic injection [103,104]. A promising novel platform that combines immunogenicity with a lack of reactogenicity are virus-like particles-based vaccines that are able to induce high protective allergen-specific IgG titers while reducing the allergenicity of the allergen [105,106]. The immunogenicity of exosomes could be harnessed in a similar fashion and could represent a physiological nanoparticle. Hence, exosomes packaged with allergens or p-MHC complexes could be loaded with other components, such as co-stimulatory molecules or mRNA/miRNA, a concept that has been put forth with DC-derived exosomes [61]. Potentially, the goal of inducing A study showed that TH1-like immune responses could be achieved by packaging native antigens into exosomes [107]. The ability to induce TH1-like responses using exosome-based vaccines has been reported in multiple studies showing that they can induce IFN-γ, TNF-α T cell responses and boost IgG antibody responses [108,109,110]. In conclusion, a vaccination approach based on exosomes that promote protective IgG responses which inhibit IgE effector function could be an interesting strategy in allergy immunotherapy (Figure 4).

### 6.4. Using Exosomes to Modulate IgE-Facilitated Antigen Presentation

**Figure 5:**
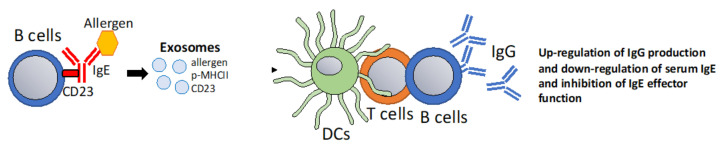
B cell-derived exosomes that modulate IgE-Facilitated Antigen Presentation. It has been proposed, that the low affinity IgE receptor CD23 can sort its ligands into exosomes [122]. Potentially, B cell mediated sorting of IgE-allergen complexes into exosomes via CD23 could be harnessed to promote T cell and IgG responses. Additionally, CD23 ligation in B cells has the effect of down-regulating IgE responses [113].

IgE in complex with antigen (Ag) forms IgE-Ag immune complexes that are highly immunogenic and lead to a CD4+ T cell and antibody responses in a process that has been termed IgE-facilitated antigen presentation IgE-FAP [111,112]. The detailed mechanism of this immune regulation mechanism and how it regulates allergic disease is still a matter of investigation. IgE-FAP involves the low-affinity receptor CD23 expressed in B cells which can shuttle IgE-Ag complexes in the follicles where they transfer antigen to dendritic cells [113,114]. Additionally, CD23 negatively regulates IgE levels in the serum in a negative feedback fashion by inhibition of IgE responses and down-regulation of serum IgE levels [115,116,117,118]. There are two CD23 isoforms that differ in their intracellular signaling sequence. CD23a internalizes IgE-Ag complexes via endocytosis whereas CD23b leads to phagocytic uptake [119]. DCs expressing CD23b degrade antigens while in B cells that mainly express CD23a, the antigen is protected and co-recycled with MHCII to the cell surface [120,121]. Independently, a study showed that in B cells, CD23-mediated internalization results in ADAM-10 dependent sorting into exosomes [122]. ADAM-10 is the principle sheddase of CD23 and is highly expressed in Golgi-derived vesicles, suggesting that CD23 shedding and/or release in exosomes requires endocytosis to allow ADAM10 to bind and to cleave CD23 [123,124,125,126]. Furthermore, it was shown that in B cells, exosome sorting of CD23 is co-regulated by engagement of the adrenergic receptor β2AR which controls ADAM-10 expression as well as protein expression of CD23 which localizes to exosomes [127]. We and others proposed a model in which B cell-expressed CD23 recycles IgE-Ag complexes into exosomes that could carry IgE, allergen, MHCII and CD23 which are able to induce T cell proliferation and antibody responses [113,128]. In that case, exosomes derived from CD23-activated B cells could potentially be used to manipulate IgE-facilitated antigen presentation to boost T cell and antibody responses while simultaneously down-regulating serum IgE levels (Figure 5).

## 7. Discussion

The general importance of exosomes in the modulation of immune responses as well as their therapeutic value in immunopathology is widely accepted. We argue that exosomes also hold this therapeutic potential in allergic diseases, which are becoming a major global health threat and additionally represent a significant economic burden. The fact that exosome-based therapeutics, so far mainly DC or MSC derived, have reached the clinical trial stage is encouraging and could thus, also be applied in allergic disease in a similar fashion. It has to be noted that allergic diseases, as well as allergy immunotherapy, are peculiar in that they are still not completely understood from an immunological standpoint. In one way, allergies can be viewed as an overreaction of the immune system in that specific IgE is overproduced in response to a harmless antigen. In another way, allergies can be viewed as the underproduction of high-affinity, antigen-specific IgG antibodies that protect the body from IgE-mediated effector functions.

The advantage of exosomes is that they have shown the ability to modulate the immune systems in both directions. MSC-derived exosomes are widely established in suppressing inflammation and exosomes derived from tolerogenic DCs or regulatory T cells have been proposed to act in a similar fashion. In contrast, exosomes have the ability to boost immune responses like classical vaccines. The ability of exosomes to carry p-MHCII complexes and/or native antigens as well as co-stimulatory molecules can facilitate strong simultaneous stimulation of T cells and B cells. In cancer immunotherapy, many candidates that boost anti-cancer responses are currently investigated. Since both in tumors and in allergy, there is a lack of TH1-like responses, it could be speculated that some of the same candidates could also be studied in allergy. However, to our knowledge, asthma or allergies have not yet been part of such investigations.

One way or another, the development of novel therapeutics in allergic diseases is very important and the evaluation of exosomes in preclinical and clinical settings can also enhance our basic immunological knowledge about allergies. AIT, the only disease-modifying therapy for allergic diseases is well established but still far from perfect, as it is not available for all allergies, displays strong variations in success rate depending on the allergen, requires patients to undergo repeated treatment over long time periods, and often bears risks of side effects. The side effects occur since allergens, the same substances that cause the IgE-mediated overreaction by the immune system, are injected to eventually promote tolerance over time. We hypothesize that a key advantage of exosomes in allergy immunotherapy could be the shielding of allergens from IgE-sensitized FcεRI expressed by mast cells and basophils. Thus, allergen-specific immune responses could be modified with a much lower risk of triggering allergic effector cells and causing anaphylactic reactions in patients. In addition to shielding an allergen, the use of FcεRI expressing exosomes could even contribute to the systemic down-regulation of IgE levels.

In summary, even though we are still far away, we believe that exosomes driving tolerance or deviating immune responses could represent a future tool for the optimization of allergy immunotherapy. However, it needs to be clarified that the here-described mechanistic approaches to treat allergy remain very theoretical due to the novelty of the field and thus it is obvious that more studies need to be done. Nevertheless, we believe that there are potential mechanisms by which exosomes could be engineered as therapeutic agents in allergy immunotherapy.

## Data Availability

Not applicable.
